# Primary eye health care: what do young children need?

**Published:** 2022-03-01

**Authors:** Milka Mafwiri, Aeesha NJ Malik

**Affiliations:** 1 Senior Lecturer & Consultant Ophthalmologist: Department of Ophthalmology, Muhimbili University of Health and Allied Sciences, Dar es Salaam, Tanzania.; 2 Consultant Ophthalmic Surgeon & Clinical Research Fellow: International Centre for Eye Health, London School of Hygiene & Tropical Medicine, London, UK.

**Integrated primary eye health care is vital for the early detection, referral, and management of eye conditions that affect young children.**

**Figure:**
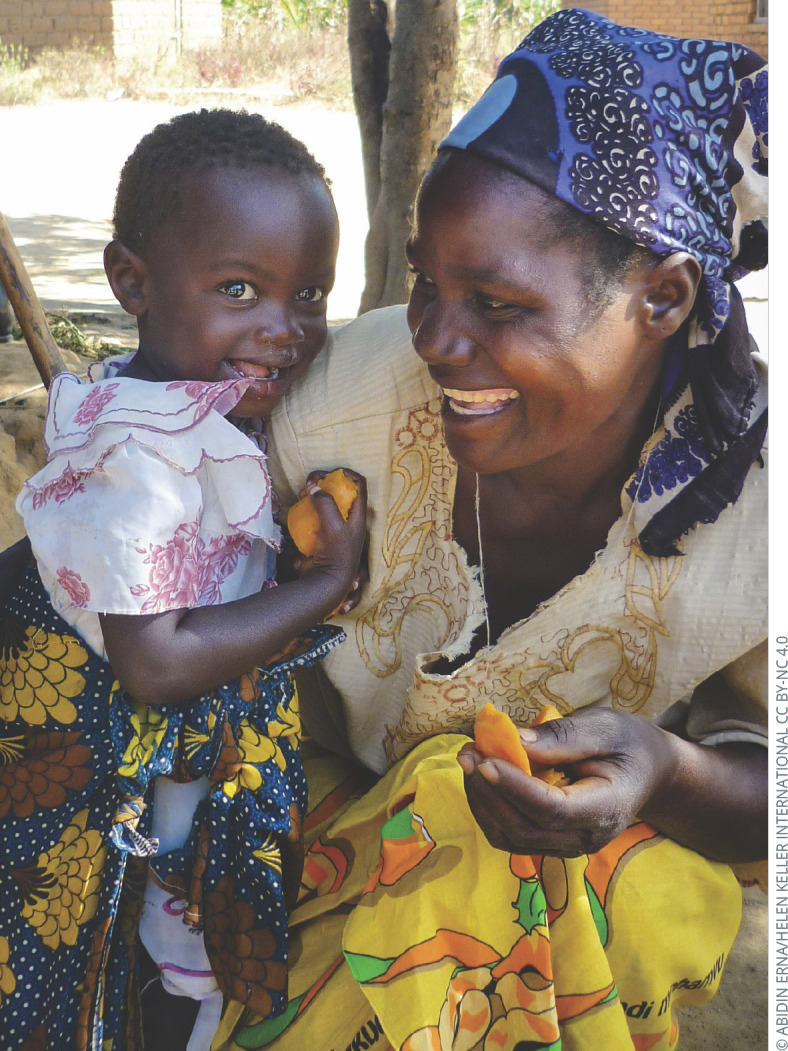
Primary health care workers can educate mothers about a healthy diet and facial hygiene.

In 2020, the Vision Loss Expert Group estimated that there are 70.2 million children aged 0–14 years who are vision impaired, 1.44 million of whom are blind.^1^ These figures are greater in countries in South Asia and sub-Saharan Africa due to the higher estimated prevalence of blindness and vision impairment, and the larger child populations in these regions.

Studies indicate that most blind children are either born blind from congenital conditions or become blind before the age of 5 years from acquired conditions. Approximately 40–50% of these causes are avoidable, meaning that they can be prevented or treated. Timely intervention is extremely important in eye conditions with early onset, as the longer the delay before treatment, the greater the negative impact on not only the child's life-long vision, but also on their motor, cognitive, social, and emotional development. For example, cataract, glaucoma, and retinoblastoma can be present at birth or develop during infancy or early childhood.

In most countries, primary health care workers are in contact with mothers and children aged 0–5 years in the antenatal period, when mothers attend primary health facilities for monitoring of pregnancy, and in primary health centres, where children regularly attend for growth monitoring, vitamin A supplementation, and immunisations. Primary health care workers are, therefore, very well placed to identify and appropriately refer children with eye conditions; however, they need the knowledge, skills, and equipment to do so, which is often limited.

**What can primary health care workers do?**

1. **Promote healthy eyes.** Encourage mothers to attend antenatal clinics where they can be screened and treated for sexually transmitted diseases to prevent ophthalmia neonatorum. Educate mothers about a healthy diet and facial hygiene.
2. **Prevent eye diseases.** Give ocular prophylaxis (antibiotics) immediately after birth to prevent ophthalmia neonatorum. Promote and deliver vitamin A supplementation to children aged from 6 months to 5 years (where this is advised) and offer measles immunisation of infants to prevent corneal blindness.
3. **Treat eye diseases.** Treat common eye diseases such as conjunctivitis.
4. **Refer complex diseases.** Identify and refer children with serious conditions such as corneal ulcers, cataract, and retinoblastoma
5. **Rehabilitate children with incurable conditions.** Encourage parents and children to wear their spectacles, if needed.

## Components of primary eye health care for children

The aim of primary eye health care for children is to prevent and reduce blindness in children. The World Health Organization's ‘ten key activities for healthy eyes in children’ provide a clear blueprint for what needs to be done at primary care level.^2^ The activities address both prevention and active management of eye diseases in children. The activities fall into three categories:

1. Health promotion for mothers (e.g., breast feeding, face washing, and good nutrition).
2. Ensuring high coverage of specific preventive measures (e.g., vitamin A supplementation, measles immunisation, and ocular prophylaxis to prevent ophthalmia neonatorum).
3. The detection and referral of children with treatable eye conditions (e.g., cataract, glaucoma, corneal ulcers, and retinoblastoma).

Primary health workers need to have skills in health promotion and taking a history, and they must know how to instil eye medication and how to perform a simple eye examination and red reflex test so that they can recognise conditions which must be referred to an eye care worker. These include:

- White pupils or an abnormal red reflex
- Abnormally small or large eyes
- Serious trauma
- Red painful eyes
- Concerns about a child's vision expressed by the parent or carer.

## Integration of primary eye health care into child health programmes

Integrating eye health into child health would mean that all primary health care workers caring for children would be trained to prevent, detect, and refer eye conditions in children. Many of the key interventions are already components of child health programmes, such as measles immunisation, but there would be some new essential clinical skills for primary health care workers to learn, such as red reflex testing and basic examination of children's eyes using a torch.

The benefit of primary eye health care for children as an integral component of child health services is potentially enormous, as it would increase access to eye health promotion and preventive measures, as well as screening and treatment, to all children.^3^ The case study from Bangladesh published elsewhere in this issue, and a programme in Tanzania,^4^ show how engaging ministries of health led to policy change, which in turn led to the inclusion of eye health in the World Health Organization's Integrated Management of Childhood Illness (IMNCI) programme.4 These demonstrate how working with governments can lead to positive change on a very large scale.
